# Mini review of first-in-human integrin αvβ6 PET tracers

**DOI:** 10.3389/fnume.2023.1271208

**Published:** 2023-10-09

**Authors:** Richard H. Kimura, Andrei Iagaru, H. Henry Guo

**Affiliations:** Department of Radiology, Stanford University School of Medicine, Stanford, CA, United States

**Keywords:** integrin αVβ6, PET, peptide, cancer, long covid, IPF

## Abstract

This mini review of clinically-evaluated integrin αvβ6 PET-tracers reveals distinct differences in human-biodistribution patterns between linear peptides, including disulfide-stabilized formats, compared to head-to-tail cyclized peptides. All PET tracers mentioned in this mini review were able to delineate disease from normal tissues, but some αvβ6 PET tracers are better than others for particular clinical applications. Each αvβ6 PET tracer was validated for its ability to bind integrin αvβ6 with high affinity. However, all the head-to-tail cyclized peptide PET-tracers reviewed here did not accumulate in the GI-tract, in striking contrast to the linear and disulfide-bonded counterparts currently undergoing clinical evaluation in cancer, IPF and long COVID. Multiple independent investigators have reported the presence of β6 mRNA as well as αvβ6 protein in the GI-tract. Currently, there remains further need for biochemical, clinical, and structural data to satisfactorily explain the state-of-the-art in human αvβ6-imaging.

## Introduction

The integrin family of transmembrane receptors consist of 24 members that perform many essential regulatory functions both inside and outside of the cell (1, 2). One member of high clinical interest is integrin αvβ6, an epithelially-restricted receptor that plays a role in wound-healing by promoting deposition of collagen via the TGFβ/SMAD pathway (3). Chronic overexpression of integrin αvβ6 occurs in many different cancers and in idiopathic pulmonary fibrosis (IPF) (4). The presence of integrin αvβ6 in disease is associated with high-morbidity and poor-survival (5–7). Integrin αvβ6 activates TGFβ by physically-removing a latency associated peptide from TGFβ complex *in situ* (8). TGFβ is a pleiotropic growth factor that is expressed by many cell types, stored in the stroma, and found ubiquitously throughout the body (9). Recently, the integrin αvβ6-TGFβ axis has been shown to repress the activity of CD8^+^ T-cells through a SOX4 mediated immunoregulatory pathway in triple negative breast cancer (10). This mini review examines integrin αvβ6 PET tracers that have been evaluated in several human cancers including pancreatic cancer, lung cancer, colon cancer, liver cancer, head and neck cancer, and metastatic disease. Integrin αvβ6 PET is also useful for chronic lung fibrosis such as IPF, and for visualizing the extent of lung injury in COVID-19 (11, 12).

Efforts to image and treat disease through the integrin αvβ6-TGFβ axis have led to the development of a wide range of ligands including small molecules and peptides, small protein domains, antibody fragments and monoclonal antibodies. For PET imaging, the smaller ligands are often preferred due to their favorable pharmacokinetic properties, robust and stoichiometric synthesis routes, availability of diverse chemical modification schemes, ease of handling and lower cost compared to biologics. Several successful strategies have been used to develop the leading candidates highlighted in this mini review. All first-in-human integrin αvβ6 PET tracers have demonstrated clinical utility in phase 0–2b clinical trials. Strengths, weaknesses, similarities, and differences will be explored for each.

**Figure 1:**
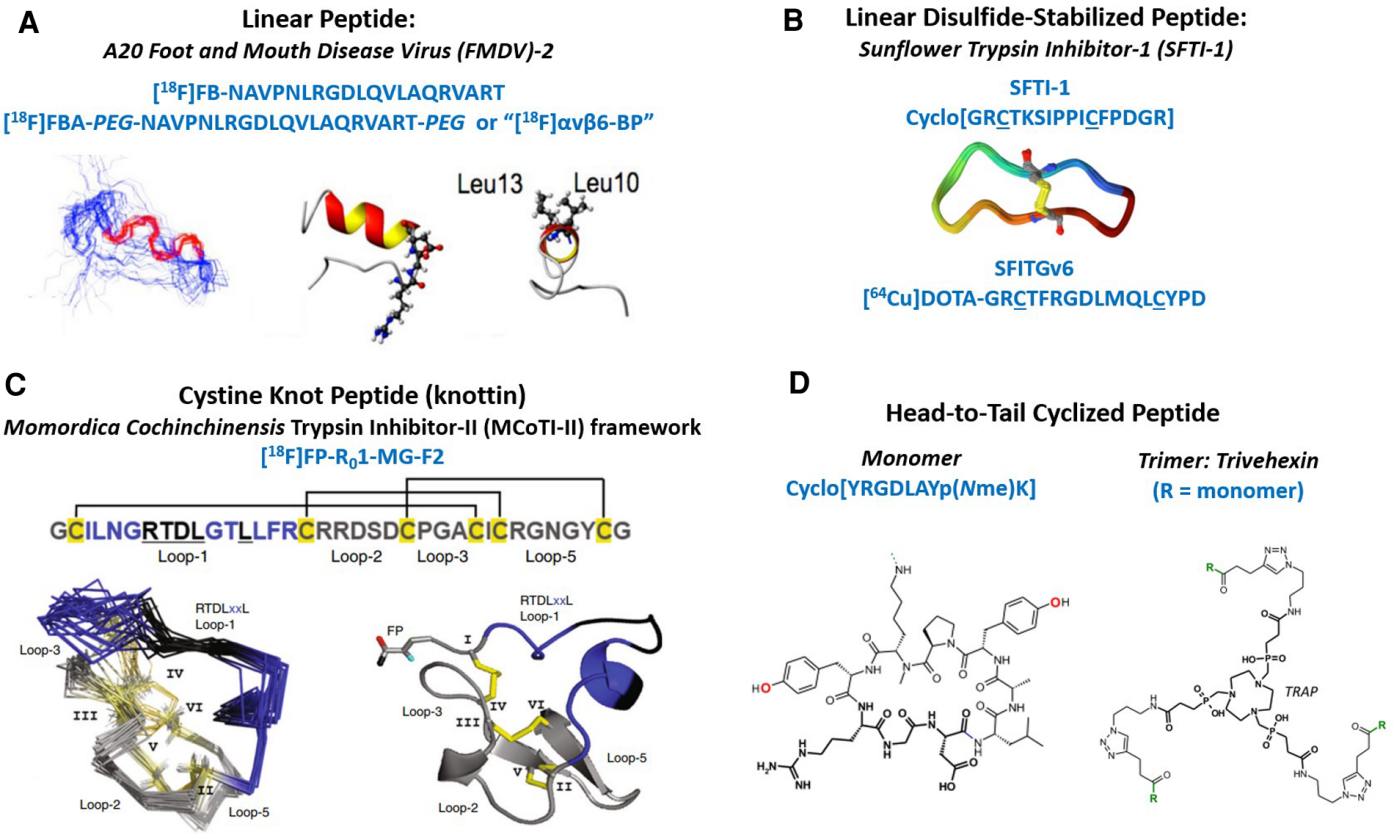
Structural overview of integrin αvβ6 PET tracers currently in clinical trials. (**A**) Primary structure of the 20 amino acid linear peptide (A20FMDV2) derived from the VP1 protein of the Foot and Mouth Disease Virus. One variant was PEGylated prior to radiolabeling. Both peptides were radiolabeled with an [^18^F]fluorobenzoyl group. Shown on the left (below) is a 40-member ensemble of the peptide backbone obtained by NMR spectroscopy (13). The bonds colored in red show the location of the DLXXL motif. The center ribbon diagram shows a side-view of the structure that was closest to the mean, where the ball-and-stick portion contains the RGD residues. The ribbon diagram on the right is an end-on view where the Leu residues are shown in ball-and-stick format. (**B**) Primary structure of cyclic SFTI-1 (1JBL), three-dimensional structure of SFTI-1, and linear disulfide-stabilized loop, SFITGv6, containing the integrin αvβ6 recognizing element situated between the cysteine residues, which form the disulfide bond (14, 15). (**C**) Primary Structure of [^18^F]FP-R_0_1-MG-F2 containing the RTDLXXL integrin binding activity in loop-1 of the MCO-TI-II framework (4). Cysteine residues, I-VI, are shown in yellow with disulfide bonds forming several looped regions. Below left, is the ensemble of NMR structures of the parent peptide R_0_1-MG, and on the right is the crystal structure of the [^19^F]fluoropropyl-labeled knottin. (**D**) Primary structure of the head-to-tail cyclic-nonapeptide monomer used to make Trivehexin, the trimer (16). Below are the chemical structures of the monomer and trimer built around the multifunctional chelator called TRAP.

The initial group of integrin αvβ6 PET tracers currently undergoing clinical evaluation can be represented by five categories: (1) linear peptide, (2) linear disulfide-bonded peptide loop, (3) cystine knot peptide (knottin), (4) head-to-tail or backbone-cyclized peptide, and (5) multimers of cyclic peptides (Figure 1). The A20FMDV2 PET tracers utilize a linear 20 mer peptide sequence derived from the VP1 capsid protein of foot and mouth disease virus (FMDV) that uses integrin αvβ6 to infect the host (13). Disulfide-stabilized looped peptides inspired by Sunflower trypsin inhibitor-1 (SFTI-1) incorporate a single disulfide bond to constrain the ends of integrin αvβ6-binding sequences discovered by phage display, or short sequences (8-mers) derived from natural endogenous sources such as extracellular matrix proteins and growth factors (14, 17). Cystine knot peptides (knottins) inspired by *Momordica Cochinchinensis* trypsin inhibitor-II (MCoTI-II) are approximately 30–40 amino acids long and are stabilized by three disulfide-bonds arranged in a topologically-knotted configuration. Knottins can be engineered by directed evolution using a yeast surface display system to generate single-digit nanomolar integrin binders (18, 19). Head-to-tail cyclized peptides refer to short amino acid sequences (9 mers) rationally-designed and optimized to selectively recognize specific RGD-integrins such as integrin αvβ6 (20, 21). Finally, multimeric versions of cyclic-peptide monomers have been made available by novel one-pot click chemistry (22).

## Subsections

### *In vivo* performance highlights of the major classes of integrin αvβ6 PET tracers currently under clinical evaluation

#### Linear peptide

All A20FMDV2-derived PET tracers currently under clinical evaluation were labeled with a fluorobenzoyl group at the N-terminus. The initial human biodistribution and safety study showed notable retention of [^18^F]FB-A20FMDV2 in the GI-tract and liver as shown in the report by Keat et al. (23). [^18^F]FB-A20FMDV2 was subsequently used to quantify integrin αvβ6 expression in healthy vs. fibrotic lungs in the PETAL study (24). Uncorrected (for lung tissue density) mean standardized uptake values (SUVs) of [^18^F]FB-A20FMDV2 were about ∼1 in IPF lungs, and ∼0.5 in healthy lungs (24). The authors also reported an SUV_max_ of ∼3 in IPF lungs (25). [^18^F]FB-A20FMDV2 was next used to confirm target engagement in the lungs of IPF patients following a single dose of a novel inhaled αvβ6 inhibitor. Imaging results suggest αvβ6-PET might accurately identify patients who would benefit from αvβ6-targeted therapies (26). Finally, in a study by Saleem et al., [^18^F]FBA-A20FMDV2 PET was used to image lung fibrosis in lung cancer patients following pulmonary radiation therapy (27). Uncorrected mean SUVs were 0.93 ± 0.6 in irradiated lungs compared to 0.56 ± 0.21 in healthy lungs. The uptake of [^18^F]FBA-A20FMDV2 appears to be relatively high in the liver and stomach.

To address off-target accumulation of the A20FMDV2-derived PET tracers, Hausner at al. modified A20FMDV2 with a polyethylene glycol (PEG) linker prior to radiolabeling with the fluorobenzoyl moiety. This PET tracer was named [^18^F]αvβ6-BP and it was shown to detect a lung nodule (SUV_max_ = 5.2) and a metastatic-lesion at the iliac wing (SUV_max_ = 13.5) in a lung cancer patient (28). [^18^F]αvβ6-BP was also used detect breast and colon cancer as well as metastatic disease in the brain, liver, and lung (28). Finally, patchy moderate uptake of [^18^F]αvβ6-BP was found in the lungs of a COVID-19 patient two months after the initial infection (12). Compared to [^18^F]FB-A20FMDV2 described above, uptake of [^18^F]αvβ6-BP appears to be much lower in the liver compared to the non-PEGylated version. This might be attributed to increased hydrophilicity of the PET tracer by PEGylation. However, notable GI-tract uptake was also found for [^18^F]αvβ6-BP (28).

#### Disulfide-stabilized loop

The SF-X (SF = sunflower, X = unique sequences) structure was inspired by study of a serine protease sunflower trypsin inhibitor-one (SFTI-1), a 14 amino acid backbone-cyclized peptide with a single disulfide-bond across the middle forming two opposing loops (29). PET-tracers inspired by this format are, thus-far, linear peptides stabilized by a disulfide bond between the N- and C-terminus cysteine residues, or half of the SFTI-1 structure. Candidates of the SF-X design were approached by two parallel methods.

A phage-display effort yielded lead candidate SFITGv6 (FRGDLMQL), where the K_D_ = 14.8 nM for integrin αvβ6 as measured by surface plasmon resonance (14, 30). The first PET/CT scans of head-and-neck squamous cell carcinoma (HNSCC) and non-small cell lung cancer (NSCLC) patients found accumulation of [^68^Ga]-GaDOTA-SFITGv6 specifically in tumors (14). In contrast, [^18^F]FDG accumulation was detected not-only in tumors, but also in inflammatory lesions in both patients (14). In addition, significant uptake of [^68^Ga]Ga-DOTA-SFITGv6 occurs in the GI-tract, but not in the liver (14, 17). In a second comparative study, [^68^Ga]Ga-DOTA-SFITGv6 demonstrated clinical utility and image quality that was comparable to [^18^F]FDG in detection of NSCLC and metastatic disease to the brain as well as regional lymph nodes as shown by Flechsig et al. (30). SUV_max_ of 7.5 was found in a histologically proven primary tumor (30). Metastatic lesions were also easily detected.

In a parallel discovery effort, sequences that would form the active disulfide-stabilized loop were derived from human RGD-containing proteins such as fibronectin (SFFN1), tenascin C (SFTNC), vitronectin (SFVTN), and latency associated peptides 1 (SFLAP1) and 3 (SFLAP3) (17). Cysteine residues were coupled to the N- and C- termini and allowed to form a disulfide bond. SFLAP3 (GGRGDLGRL) demonstrated superior performance in cell binding assays and was subsequently advanced to clinical trials. PET/CT scanning of a HNSCC patient showed accumulation of [^68^Ga]Ga-DOTA-SFLAP in the primary tumor (SUV_max_ = 5.1) and in corresponding lymph node metastases (SUV_max_ = 4.1) (17). In contrast to the unmodified A20FMDV2 based PET tracers described above, significant uptake of SFTI-1 inspired peptides occurred in the GI-tract, but not in the liver.

#### Cystine knot peptide (knottin)

**Figure 2:**
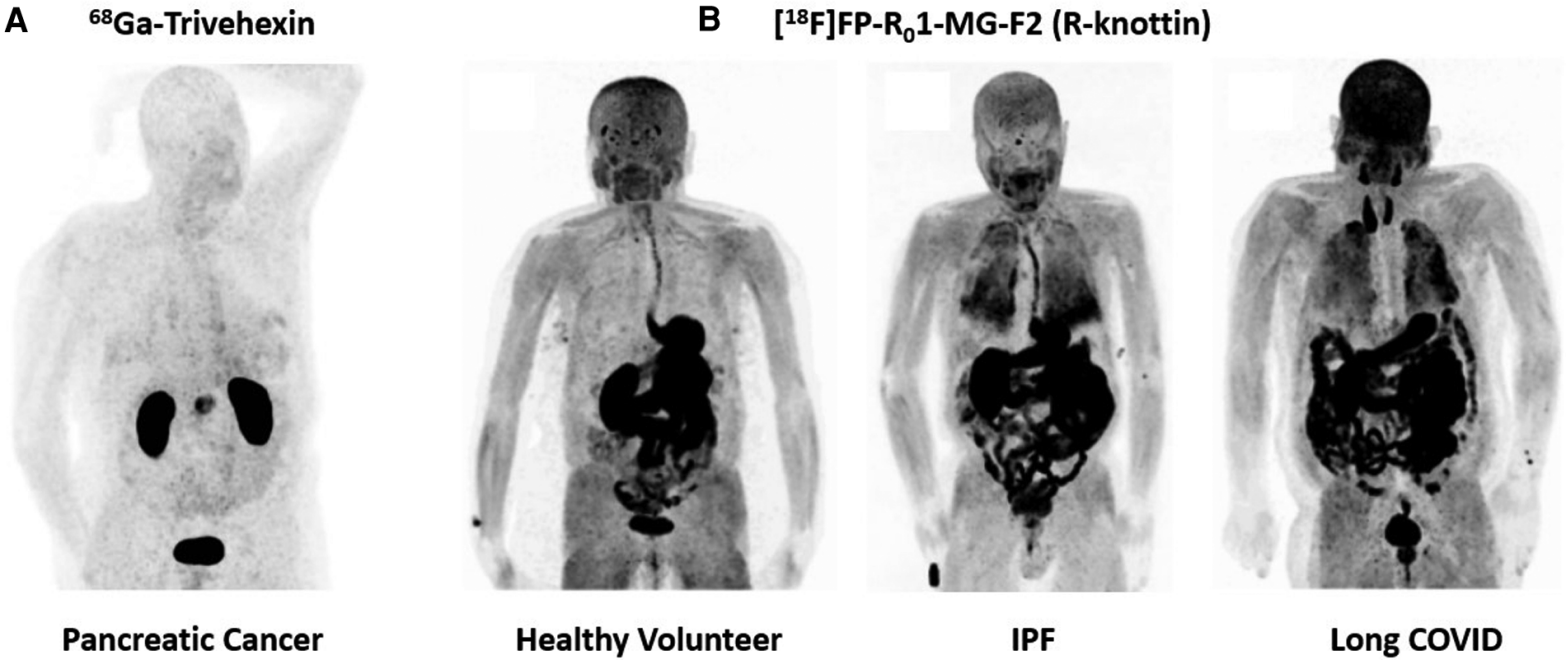
Maximum intensity projection (MIP) PET images of integrin αvβ6 PET tracers. (**A**) Detection of pancreatic cancer ^68^Ga-Trivehexin, which performs exceptionally well in the abdominal region (16). (**B**) Evaluation of [^18^F]FP-R_0_1-MG-F2 in a healthy volunteer, an IPF patient and a long COVID patient (11). This PET tracer performs well in identifying fibrotic interstitial lung disease.

[^18^F]FP-R_0_1-MG-F2 is a knottin PET tracer that was engineered to bind integrin αvβ6 with high affinity (K_D_/IC_50_∼1nM) (31). This PET tracer was built into the *Momordica cochinchinensis* Trypsin Inhibitor-II (MCoTI-II) framework and therefore contains a relatively high percentage of arginine residues compared to other inhibitor cystine knots of this family (31–36). The high arginine content of the MCo-TI-II scaffold produced pharmacokinetic properties that benefit molecular imaging applications as PET and SPECT (31). In head-to-head comparisons, the arginine-rich knottins (R-knots) outperformed serine-rich knottins (S-knots) and glutamic acid-rich knottins (E-knots) as PET agents evaluated in living animals (31). Arginine residues enhanced uptake of the PET tracer in all tissues while maintaining robust disease-to-normal tissue ratios (31). Thus, the R-knot designated R_0_1-MG was N-terminus labeled with a fluropropyl moiety and advanced to clinical trials (4). In a pilot clinical study, [^18^F]FP-R_0_1-MG-F2 produced SUV_mean_ of ∼6 in a pancreatic tumor (4). The same primary tumor imaged with [^18^F]FDG resulted in an SUV_mean_ of∼4. [^18^F]FP-R_0_1-MG-F2 also demonstrated high uptake in the lungs of IPF patients due to chronic over-expression of integrin αvβ6, which promotes lung fibrosis (4). In contrast, lung uptake was very low in healthy individuals (Figure 2).

The same parent peptide, R_0_1-MG, was next labeled with [^68^Ga]Ga-NODAGA- at the N-terminus. In a different pancreatic cancer patient, [^68^Ga]Ga-NODAGA-R_0_1-MG produced SUV_mean_ of 4.4 (4). [^68^Ga]Ga-NODAGA-R_0_1-MG also clearly-detected cervical cancer and lung cancer (4) Next, 15 pancreatic cancer patients were imaged with [^18^F]FP-R_0_1-MG-F2. The mean ± SD SUV_max_ = 12.2 ± 4.3 at 75 min after intravenous administration of the tracer (37). Metastatic disease was detected in the lymph nodes (mean ± SD SUV_max_ = 5.9 ± 3.2), lungs (mean ± SD SUV_max_ = 3.5 ± 1.8), liver (mean ± SD SUV_max_ = 6.9 ± 3.7) and peritoneum (mean ± SD SUV_max_ = 6.3 ± 2.6) (37). Finally, evaluation of [^18^F]FP-R_0_1-MG-F2 in a long COVID patient demonstrated similar lung uptake to IPF patients, where the typical SUV_max_∼8–10 (11) (Figure 2). Like the linear peptide and the disulfide-stabilized loops described above, the knottin PET tracer was also taken-up in large quantities by the GI-tract including the esophagus, stomach, small intestines, large intestines, and colon (4). However, liver uptake was very low, which could bode well for detection of fibrotic liver disease.

#### Cyclic peptide monomers and trimers

Head-to-tail cyclization of small RGD containing peptides has successfully led to the development of clinical products designed to image and treat cancer (38). Early studies by Kessler and colleagues explored integrin binding properties of many novel cyclic-RGD containing variants (38). Recent efforts led to the development of a cyclic-nonapeptide that binds integrin αvβ6 with picomolar affinity (20). Half maximal inhibitory concentration (IC_50_) values were determined using a solid phase cell adhesion assay as described by Frank et al. (39). Trimerization of the lead compound through a multi-functional chelator led to the development of a product called Trivehexin (16). Gallium-labeled Trivehexin yielded IC_50_ values of 47 pM for integrin αvβ6, 6.2 nM for integrin αvβ8, 2.7 nM for integrin αvβ3 and 22 nM for integrin α5β1 (16). Remarkably, first-in-human studies found that Trivehexin did not accumulate in GI-tract as shown in Quigley et al. (16, 40). Indeed, this *in vivo* biodistribution profile bodes well for detection of pancreatic cancer compared to the other integrin αvβ6-based PET tracer described above, all of which accumulate to high levels in the GI-tract, and thus can obstruct signals from pancreatic cancers. The favorable biodistribution of Trivehexin also suggests its clinical potential as a theranostic agent for targeted treatment of cancer (Figure 2).

Feng et al. also described the development of a backbone-cyclized octameric peptide. BxPC3 cell-based competition binding assays were conducted between [^64^Cu]Cu-DOTA-cycratide and the test compounds (21). The authors report IC_50_ values∼20 nM for the cycratide-derivatives. Figure 4 of Feng et al. showed that a significant fraction of the activity remains in the blood compartment at 60 min after administration of the PET tracer. Interestingly, like the cyclic-nonapeptides described above, cycratide PET tracers also did not accumulate to high levels in the GI-tract. This first-in-human study showed that [^68^Ga]Ga-DOTA-cycratide (SUV_max_∼4.9) was able to detect primary disease in the pancreas. Similarly, [^18^F]FDG SUV_max_∼6.9 (21).

#### Findings from this mini review

All the PET tracers surveyed in this mini review were able to detect different types of cancer. For pancreatic cancer, small backbone-cyclized peptides, Trivehexin and cycratide, demonstrated potentially-superior performance over the reviewed-set of linear peptides and disulfide-stabilized loops due to biodistribution in the GI-tract. For chest imaging, knottins PET tracers based on the R_0_1-MG peptide performed particularly well in assessing lung disease in IPF and Long COVID due to high uptake in disease tissues (Figure 2).

Currently, the reason is unknown for the large differences in PET-signal in the GI-tract between backbone-cyclized peptides (low-uptake) and linear peptides (high-uptake), particularly when all binders were validated for their ability to recognize integrin αvβ6. One hypothesis is that these molecules cross-react with different subgroups of closely-related RGD-integrins so that these differences may be due to the binder's particular specificity profile for the highly conserved RGD-integrins. Small cyclic peptides may dock into the RGD-binding pocket differently than the linear peptides or disulfide-stabilized loops. Currently, there is insufficient experimental data to satisfactorily explain the findings we have described. An answer to this question could have a significant impact on design strategies for next-generation integrin binders.

#### Expression of integrin αvβ6 in the GI-tract

Evidence of integrin αvβ6 expression in the GI tract has been shown in both human and non-human studies. Analysis of the human tissue-specific expression by genome-wide integration of transcriptomics and antibody-based proteomics indicated the presence of both β6 mRNA and protein in many parts of GI-tract, such as the stomach, duodenum, small intestine, and colon (41). Data from this study is available in a searchable database through the National Center for Biotechnology Information (www.ncbi.nlm.nih.gov). Similar data can also be located through The Human Protein Atlas (www.proteinatlas.org). Koivisto et al. reviewed literature about expression of integrin αvβ6 in human and animal models, and provides many references on studies of the GI-tract (42). Kimura et al. demonstrated positive stomach and small bowel β6 expression using immunohistochemistry, with strong anti-β6 antibody-staining revealing the presence of significant quantities of the β6 protein on the luminal side of the stomach and intestine (4). Feng et al. found expression of integrin αvβ6 in intestinal epithelial cells of patients with inflammatory bowel disease (IBD) (43). Xie et al. found elevated ITGB6 mRNA expression in IBD patients' intestinal specimens (44). Integrin αvβ6 contributes to the development of intestinal fibrosis via the FAK/AKT signaling pathway (44). Kuwada et al. identified an anti-integrin αvβ6 autoantibody in patients with ulcerative colitis (45). Rydell et al. claim that measurement of serum IgG anti-integrin αvβ6 autoantibodies is a promising tool in the diagnosis of ulcerative colitis (46). Brown et al. have demonstrated integrin αvβ6 expression in several subsections of the sheep GI-tract (47). Strong endogenous αvβ6 expression was also detected by immunohistochemical analysis of murine stomach, and moderate expression was found in the duodenum, ileum, and colon (48). Yu et al. claim that integrin αvβ6 is required for maintaining the intestinal epithelial barrier (49).

## Discussion

### Lessons from the initial integrin αvβ6 PET imaging studies in humans

The first wave of integrin αvβ6 PET tracer to enter clinical trials consisted entirely of small peptides and peptidomimetics, which produced sufficient uptake in disease tissues that enabled clear delineation from healthy tissues. These integrin αvβ6 PET tracers detected diseases such as cancer, IPF and long COVID lung injury. Interestingly, two broad categories of αvβ6 PET tracer have emerged—those that accumulated to high levels in the gut and those that did not. All non-backbone-cyclized αvβ6 PET tracers reviewed here produced high gut uptake, in contrast to the backbone-cyclized αvβ6 PET tracers, which avoided the gut. Since integrin αvβ6 has been shown to be highly expressed in the GI-tract, these findings reveal a gap in our understanding about some aspects of integrin-binding by exogenous ligands in human systems. This is particularly important because uptake of αvβ6 PET tracers by abdominal organs can obscure detection of some cancers and preclude their use as theranostics due to off-target dosing of healthy tissue.

Trivehexin is reported to be ∼100 × more selective for integrin αvβ6 (K_D_ = 47 pM) over integrin αvβ8 (K_D_ = 6.2 nM). These favorable binding properties resulted in part from tyrosine-substitution, which was used to enhance binding affinity and specificity. Tyrosine and tyrosine-like molecules are especially good at mediating binding affinity due to favorable electrostatic potential, thermodynamic cost and fitment into binding sites (50). Furthermore, Notni and colleagues used trimerization by way of a chelator to leverage the effects of avidity to further enhance binding affinity for PET imaging (16). The trimeric construct performed especially well in detecting pancreatic cancer. Importantly, high uptake in tumors, coupled with low background in the GI-tract bodes well for theranostic applications (Figure 2).

The presence of arginine residues in a PET-tracer increases uptake by all tissues, an unexpected pharmacokinetic property first discovered in mouse models of cancer (31). In human clinical trials, arginine-rich knottins (R-knottins) were taken up by disease tissues to a greater degree compared to other classes of PET tracers. In preclinical models, R-knottins were evaluated for their PET-endpoints in side-by-side comparisons against other knottin scaffolds, small peptides, antibody fragments and various other platforms presenting the RXDLXXL master key code. We hypothesized that the SUV-boost imparted by arginine residues would be valuable for high performance molecular imaging. The first-in-human PET images of IPF and Long COVID patients' lungs are the first clinical results demonstrating *in vivo* benefit of the arginine-loading strategy (Figure 2). Indeed, high fractions of basic amino acids naturally occur in a large subgroup within the family of cystine knot trypsin inhibitors (31).

Integrin-based PET will continue to evolve through development of bio-activities that selectively recognize closely-related RGD-integrin family members that are uniquely-expressed in various disease states, but also in normal organs and tissues. Integrin family members such as αvβ1, αvβ6 and αvβ8 are highly conserved in primary structure so that their binding sites are almost identical in electrostatic potential, molecular-shape, and other surface-interface characteristics (51). One challenge that currently drives the field is to produce highly-selective ligands that can distinguish between these and other integrin subtype in order to image and treat disease with greater precision. The first group of clinically-evaluated integrin αvβ6 PET tracers, reviewed here, collectively-represent one step towards our goals.

## References

[1] Ludwig BS Kessler H Kossatz S Reuning U. RGD-binding integrins revisited: how recently discovered functions and novel synthetic ligands (Re-)shape an ever-evolving field. Cancers (Basel). (2021) 13(7):1711. 10.3390/cancers13071711 33916607 PMC8038522

[2] Desgrosellier JS Cheresh DA. Integrins in cancer: biological implications and therapeutic opportunities. Nat Rev Cancer. (2010) 10(1):9–22. 10.3390/cancers13071711 20029421 PMC4383089

[3] Verrecchia F Chu ML Mauviel A. Identification of novel TGF-beta/smad gene targets in dermal fibroblasts using a combined cDNA microarray/promoter transactivation approach. J Biol Chem. (2001) 276(20):17058–62. 10.1074/jbc.M100754200 11279127

[4] Kimura RH Wang L Shen B Huo L Tummers W Filipp FV Evaluation of integrin alphavbeta6 cystine knot PET tracers to detect cancer and idiopathic pulmonary fibrosis. Nat Commun. (2019) 10(1):4673. 10.1038/s41467-019-11863-w 31611594 PMC6791878

[5] Munger JS Huang X Kawakatsu H Griffiths MJ Dalton SL Wu J The integrin alpha v beta 6 binds and activates latent TGF beta 1: a mechanism for regulating pulmonary inflammation and fibrosis. Cell. (1999) 96(3):319–28. 10.1016/s0092-8674(00)80545-0 10025398

[6] Saini G Porte J Weinreb PH Violette SM Wallace WA McKeever TM Alphavbeta6 integrin may be a potential prognostic biomarker in interstitial lung disease. Eur Respir J. (2015) 46(2):486–94. 10.1183/09031936.00210414 25745053

[7] Bandyopadhyay A Raghavan S. Defining the role of integrin alphavbeta6 in cancer. Curr Drug Targets. (2009) 10(7):645–52. 10.2174/138945009788680374 19601768 PMC2888263

[8] Munger JS Sheppard D. Cross talk among TGF-beta signaling pathways, integrins, and the extracellular matrix. Cold Spring Harb Perspect Biol. (2011) 3(11):a005017. 10.1101/cshperspect.a005017 21900405 PMC3220354

[9] Moses HL Roberts AB Derynck R. The discovery and early days of TGF-beta: a historical perspective. Cold Spring Harb Perspect Biol. (2016) 8(7):1–26. 10.1101/cshperspect.a021865 PMC4930926 27328871

[10] Bagati A Kumar S Jiang P Pyrdol J Zou AE Godicelj A Integrin alphavbeta6-TGFbeta-SOX4 pathway drives immune evasion in triple-negative breast cancer. Cancer Cell. (2021) 39(1):54–67. 10.1016/j.ccell.2020.12.001 33385331 PMC7855651

[11] Kimura RH Sharifi H Shen B Berry GJ. Guo HH. Alpha(v)beta(6) integrin positron emission tomography of lung fibrosis in idiopathic pulmonary fibrosis and long-COVID. Am J Respir Crit Care Med. (2023) 207(12):1633–5. 10.1164/rccm.202206-1107IM 36693032

[12] Foster CC Davis RA Hausner SH. Sutcliffe JL. Alpha(v)beta(6)-targeted molecular PET/CT imaging of the lungs after SARS-CoV-2 infection. J Nucl Med. (2020) 61(12):1717–9. 10.2967/jnumed.120.255364 32948681 PMC8679627

[13] DiCara D Rapisarda C Sutcliffe JL Violette SM Weinreb PH Hart IR Structure-function analysis of arg-gly-asp helix motifs in alpha v beta 6 integrin ligands. J Biol Chem. (2007) 282(13):9657–65. 10.1074/jbc.M610461200 17244604

[14] Altmann A Sauter M Roesch S Mier W Warta R Debus J Identification of a novel ITGalpha(v)beta(6)-binding peptide using protein separation and phage display. Clin Cancer Res. (2017) 23(15):4170–80. 10.1158/1078-0432.CCR-16-3217 28468949

[15] Korsinczky ML Schirra HJ Rosengren KJ West J Condie BA Otvos L Solution structures by 1H NMR of the novel cyclic trypsin inhibitor SFTI-1 from sunflower seeds and an acyclic permutant. J Mol Biol. (2001) 311(3):579–91. 10.1006/jmbi.2001.4887 11493011

[16] Quigley NG Steiger K Hoberück S Czech N Zierke MA Kossatz S PET/CT imaging of head-and-neck and pancreatic cancer in humans by targeting the “cancer integrin” alphavbeta6 with ga-68-trivehexin. Eur J Nucl Med Mol Imaging. (2022) 49(4):1136–47. 10.1007/s00259-021-05559-x 34559266 PMC8460406

[17] Roesch S Lindner T Sauter M Loktev A Flechsig P Müller M Comparison of the RGD motif-containing alpha(v)beta(6) integrin-binding peptides SFLAP3 and SFITGv6 for diagnostic application in HNSCC. J Nucl Med. (2018) 59(11):1679–85. 10.2967/jnumed.118.210013 29674419

[18] Kimura RH Cheng Z Gambhir SS Cochran JR. Engineered knottin peptides: a new class of agents for imaging integrin expression in living subjects. Cancer Res. (2009) 69(6):2435–42. 10.1158/0008-5472.CAN-08-2495 19276378 PMC2833353

[19] Kimura RH Levin AM Cochran FV Cochran JR. Engineered cystine knot peptides that bind alphavbeta3, alphavbeta5, and alpha5beta1 integrins with low-nanomolar affinity. Proteins. (2009) 77(2):359–69. 10.1002/prot.22441 19452550 PMC5792193

[20] Maltsev OV Marelli UK Kapp TG Di Leva FS Di Maro S Nieberler M Stable peptides instead of stapled peptides: highly potent alphavbeta6-selective integrin ligands. Angew Chem Int Ed Engl. (2016) 55(4):1535–9. 10.1002/anie.201508709 26663660

[21] Feng X Wang Y Lu D Xu X Zhou X Zhang H Clinical translation of a (68)Ga-labeled integrin alpha(v)beta(6)-targeting cyclic radiotracer for PET imaging of pancreatic cancer. J Nucl Med. (2020) 61(10):1461–7. 10.2967/jnumed.119.237347 32086242 PMC7539652

[22] Baranyai Z Reich D Vágner A Weineisen M Tóth I Wester HJ A shortcut to high-affinity ga-68 and cu-64 radiopharmaceuticals: one-pot click chemistry trimerisation on the TRAP platform. Dalton Trans. (2015) 44(24):11137–46. 10.1039/c5dt00576k 25999035

[23] Keat N Kenny J Chen K Onega M Garman N Slack RJ A microdose PET study of the safety, immunogenicity, biodistribution, and radiation dosimetry of (18)F-FB-A20FMDV2 for imaging the integrin alpha(v)beta(6). J Nucl Med Technol. (2018) 46(2):136–43. 10.2967/jnmt.117.203547 29438002

[24] Lukey PT Coello C Gunn R Parker C Wilson FJ Saleem A Clinical quantification of the integrin alphavbeta6 by [(18)F]FB-A20FMDV2 positron emission tomography in healthy and fibrotic human lung (PETAL study). Eur J Nucl Med Mol Imaging. (2020) 47(4):967–79. 10.1007/s00259-019-04586-z 31814068 PMC7075837

[25] Lukey PT Wilson FJ. Quantification of the alpha (v) beta (6) integrin by PET/CT imaging in the lungs of patients after SARS-CoV2 infection and comparison to fibrotic lungs. J Nucl Med. (2022) 63(1):166. 10.2967/jnumed.121.262342 33837070 PMC8717206

[26] Maher TM Simpson JK Porter JC Wilson FJ Chan R Eames R A positron emission tomography imaging study to confirm target engagement in the lungs of patients with idiopathic pulmonary fibrosis following a single dose of a novel inhaled alphavbeta6 integrin inhibitor. Respir Res. (2020) 21(1):75. 10.1186/s12931-020-01339-7 32216814 PMC7099768

[27] Saleem A Helo Y Win Z Dale R Cook J Searle GE Integrin alphavbeta6 positron emission tomography imaging in lung cancer patients treated with pulmonary radiation therapy. Int J Radiat Oncol Biol Phys. (2020) 107(2):370–6. 10.1016/j.ijrobp.2020.02.014 32060008

[28] Hausner SH Bold RJ Cheuy LY Chew HK Daly ME Davis RA Preclinical development and first-in-human imaging of the integrin alpha(v)beta(6) with [(18)F]alpha(v)beta(6)-binding peptide in metastatic carcinoma. Clin Cancer Res. (2019) 25(4):1206–15. 10.1158/1078-0432.CCR-18-2665 30401687 PMC6377828

[29] Luckett S Garcia RS Barker JJ Konarev AV Shewry PR Clarke AR High-resolution structure of a potent, cyclic proteinase inhibitor from sunflower seeds. J Mol Biol. (1999) 290(2):525–33. 10.1006/jmbi.1999.2891 10390350

[30] Flechsig P Lindner T Loktev A Roesch S Mier W Sauter M PET/CT imaging of NSCLC with a alpha(v)beta(6) integrin-targeting peptide. Mol Imaging Biol. (2019) 21(5):973–83. 10.1007/s11307-018-1296-6 30671741

[31] Kimura RH Teed R Hackel BJ Pysz MA Chuang CZ Sathirachinda A Pharmacokinetically stabilized cystine knot peptides that bind alpha-v-beta-6 integrin with single-digit nanomolar affinities for detection of pancreatic cancer. Clin Cancer Res. (2012) 18(3):839–49. 10.1158/1078-0432.CCR-11-1116 22173551 PMC3271184

[32] Heitz A Hernandez JF Gagnon J Hong TT Pham TT Nguyen TM Solution structure of the squash trypsin inhibitor MCoTI-II. A new family for cyclic knottins. Biochemistry. (2001) 40(27):7973–83. 10.1021/bi0106639 11434766

[33] Hernandez JF Gagnon J Chiche L Nguyen TM Andrieu JP Heitz A Squash trypsin inhibitors from Momordica cochinchinensis exhibit an atypical macrocyclic structure. Biochemistry. (2000) 39(19):5722–30. 10.1021/bi9929756 10801322

[34] Chiche L Heitz A Gelly JC Gracy J Chau PT Ha PT Squash inhibitors: from structural motifs to macrocyclic knottins. Curr Protein Pept Sci. (2004) 5(5):341–9. 10.2174/1389203043379477 15551519

[35] Cemazar M Joshi A Daly NL Mark AE Craik DJ. The structure of a two-disulfide intermediate assists in elucidating the oxidative folding pathway of a cyclic cystine knot protein. Structure. (2008) 16(6):842–51. 10.1016/j.str.2008.02.023 18547517

[36] Daly NL Clark RJ Craik DJ. Disulfide folding pathways of cystine knot proteins. Tying the knot within the circular backbone of the cyclotides. J Biol Chem. (2003) 278(8):6314–22. 10.1074/jbc.M210492200 12482862

[37] Nakamoto R Ferri V Duan H Hatami N Goel M Rosenberg J Pilot-phase PET/CT study targeting integrin alphavbeta6 in pancreatic cancer patients using the cystine-knot peptide-based (18)F-FP-R01-MG-F2. Eur J Nucl Med Mol Imaging. (2022) 50(1):184–93. 10.1007/s00259-021-05595-7 34729628

[38] Aumailley M Gurrath M Müller G Calvete J Timpl R Kessler H. Arg-Gly-Asp constrained within cyclic pentapeptides. Strong and selective inhibitors of cell adhesion to vitronectin and laminin fragment P1. FEBS Lett. (1991) 291(1):50–4. 10.1016/0014-5793(91)81101-d 1718779

[39] Frank AO Otto E Mas-Moruno C Schiller HB Marinelli L Cosconati S Conformational control of integrin-subtype selectivity in isoDGR peptide motifs: a biological switch. Angew Chem Int Ed Engl. (2010) 49(48):9278–81. 10.1002/anie.201004363 20957712

[40] Quigley NG Czech N Sendt W Notni J. PET/CT imaging of pancreatic carcinoma targeting the “cancer integrin” alphavbeta6. Eur J Nucl Med Mol Imaging. (2021) 48(12):4107–8. 10.1007/s00259-021-05443-8 34109438 PMC8484182

[41] Fagerberg L Hallström BM Oksvold P Kampf C Djureinovic D Odeberg J Analysis of the human tissue-specific expression by genome-wide integration of transcriptomics and antibody-based proteomics. Mol Cell Proteomics. (2014) 13(2):397–406. 10.1074/mcp.M113.035600 24309898 PMC3916642

[42] Koivisto L Bi J Häkkinen L Larjava H. Integrin alphavbeta6: structure, function and role in health and disease. Int J Biochem Cell Biol. (2018) 99:186–96. 10.1016/j.biocel.2018.04.013 29678785

[43] Feng BS Chen X Li P Zheng PY Chong J Cho DB Expression of integrin alphavbeta6 in the intestinal epithelial cells of patients with inflammatory bowel disease. N Am J Med Sci. (2009) 1(4):200–4. 10.4297/najms.2009.4200 22666696 PMC3364666

[44] Xie H Jiao Y Zhou X Liao X Chen J Chen H Integrin alphavbeta6 contributes to the development of intestinal fibrosis via the FAK/AKT signaling pathway. Exp Cell Res. (2022) 411(2):113003. 10.1016/j.yexcr.2021.113003 34979108

[45] Kuwada T Shiokawa M Kodama Y Ota S Kakiuchi N Nannya Y Identification of an anti-integrin alphavbeta6 autoantibody in patients with ulcerative colitis. Gastroenterology. (2021) 160(7):2383–94. 10.1053/j.gastro.2021.02.019 33582126

[46] Rydell N Ekoff H Hellström PM Movérare R. Measurement of Serum IgG anti-integrin alphavbeta6 autoantibodies is a promising tool in the diagnosis of ulcerative colitis. J Clin Med. (2022) 11(7):1881. 10.3390/jcm11071881 35407486 PMC8999661

[47] Brown JK McAleese SM Thornton EM Pate JA Schock A Macrae AI Integrin-alphavbeta6, a putative receptor for foot-and-mouth disease virus, is constitutively expressed in ruminant airways. J Histochem Cytochem. (2006) 54(7):807–16. 10.1369/jhc.5A6854.2006 16517977

[48] Saha A Ellison D Thomas GJ Vallath S Mather SJ Hart IR High-resolution in vivo imaging of breast cancer by targeting the pro-invasive integrin alphavbeta6. J Pathol. (2010) 222(1):52–63. 10.1002/path.2745 20629113

[49] Yu Y Chen S Lu GF Wu Y Mo L Liu ZQ Alphavbeta6 is required in maintaining the intestinal epithelial barrier function. Cell Biol Int. (2014) 38(6):777–81. 10.1002/cbin.10258 24677750

[50] Koide S Sidhu SS. The importance of being tyrosine: lessons in molecular recognition from minimalist synthetic binding proteins. ACS Chem Biol. (2009) 4(5):325–34. 10.1021/cb800314v 19298050 PMC2829252

[51] Wang J Su Y Iacob RE Engen JR Springer TA. General structural features that regulate integrin affinity revealed by atypical alphaVbeta8. Nat Commun. (2019) 10(1):5481. 10.1038/s41467-019-13248-5 31792290 PMC6889490

